# A Comparative Evaluation of Microleakage in Class V Composite Restorations

**DOI:** 10.1155/2014/685643

**Published:** 2014-12-25

**Authors:** Sujatha Gopal Sooraparaju, Pavan Kumar Kanumuru, Surya Kumari Nujella, Karthik Roy Konda, K. Bala Kasi Reddy, Sivaram Penigalapati

**Affiliations:** Department of Conservative Dentistry & Endodontics, MNR Dental College and Hospital, Telangana, India

## Abstract

*Aim*. To compare and evaluate the microleakage in class V lesions restored with composite resin with and without liner and injectable nanohybrid composite resin. *Materials and Methodology*. 60 class V cavities were prepared in 30 freshly extracted teeth. After etching and application of bonding agents these cavities were divided into three groups: Group A (*n* = 20)—restored with composite resin, Group B (*n* = 20)—flowable composite resin liner + composite resin, and Group C (*n* = 20)—restored with injectable composite resin. After curing all the specimens were subjected to thermocycling and cyclic loading. Specimens were stained with 0.5% basic fuchsin and evaluated for dye penetration. *Results*. Results are subjected to Kruskal Wallis and Wilcoxon test. *Conclusion*. Within the limitations of this study, none of the three materials were free from microleakage. All the three materials showed more microleakage at gingival margins compared to occlusal margins. Among all the groups G-ænial Flo showed the least microleakage at the gingival wall.

## 1. Introduction

Dentistry had always thrived to achieve biocompatible restorations that do not compromise the pulp and also maintain the dental seal. One of the significant contributions has been the development of resin-based composite technology. With the constant increase in aesthetic demands composites are the widely used restorative material [1]. Developments in filler technology and initiator systems have considerably improved composite physical properties and expanded their clinical applications. Cervical lesions are very often caused by incorrect tooth brushing and dental caries and usually have little or no enamel at the cervical margin [2]. Flowable composite resins are widely used in clinical practice and are the most common resin materials that are recommended for restoring these lesions instead of conventional resin composites because of low viscosity and good aesthetic properties [3, 4]. The major disadvantage of visible light-cured composites is polymerization shrinkage. This shrinkage can result in gap formation between the composite material and tooth structure, particularly if the restoration margin is placed in dentin or cementum [5]. Bacteria, fluids, molecules, or ions can pass through this gap between the resin composite and the cavity wall, a process called microleakage [6]. Microleakage is thought to be responsible for hypersensitivity, secondary caries, pulpal pathosis, and failure of restorations [7]. Besides pulpal irritation and secondary caries, microleakage also results in marginal discoloration. The use of a liner to act as a flexible intermediate layer between restoration and substrate has been suggested as a method of relieving the stress associated with polymerization shrinkage [8]. Flowable composites have been recommended as liners due to their low viscosity, increased elasticity, and wettability [9]. G-ænial Universal Flo is a light-cured radiopaque injectable nanohybrid composite resin with a combination of 2 types of prepolymerized resin fillers which was recently introduced and which claimed to have low modulus of elasticity and low volumetric shrinkage. The present study was aimed at comparing and evaluating the microleakage in class V lesions restored with composite resin with and without liner and injectable nanohybrid composite resin.

## 2. Methods and Methodology

**Figure 1:**
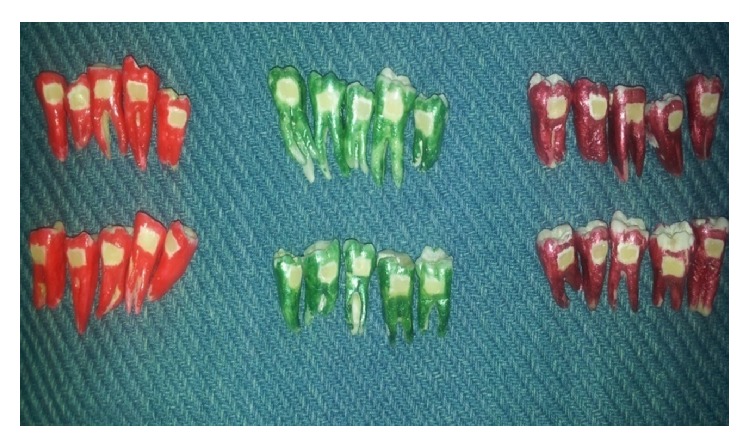
Three different groups.

Thirty recently extracted teeth for orthodontic and periodontal reasons were collected from the Department of Oral and Maxillofacial Surgery, MNR Dental College. They were checked for caries, abrasion, attrition, fluorosis, or other enamel defects, which, if present, were discarded. The teeth were cleaned of soft tissue and hard tissue debris and then class V cavity preparation on buccal and lingual surfaces was done. Cavities were prepared with standardized dimensions of height of 2 mm, width of 4 mm, and depth of 2 mm. After etching and application of bonding agents these cavities were divided into three groups (Figure 1).

*(i) Group A*. Restored with conventional nanohybrid composite resin Tetric N-Ceram using etch and rinse adhesive system.

*(ii) Group B*. 1 mm of flowable composite Tetric N-Flow applied as liner prior to the composite restoration.

*(iii) Group C*. Restored with injectable composite G-ænial Universal Flo..

**Figure 2:**
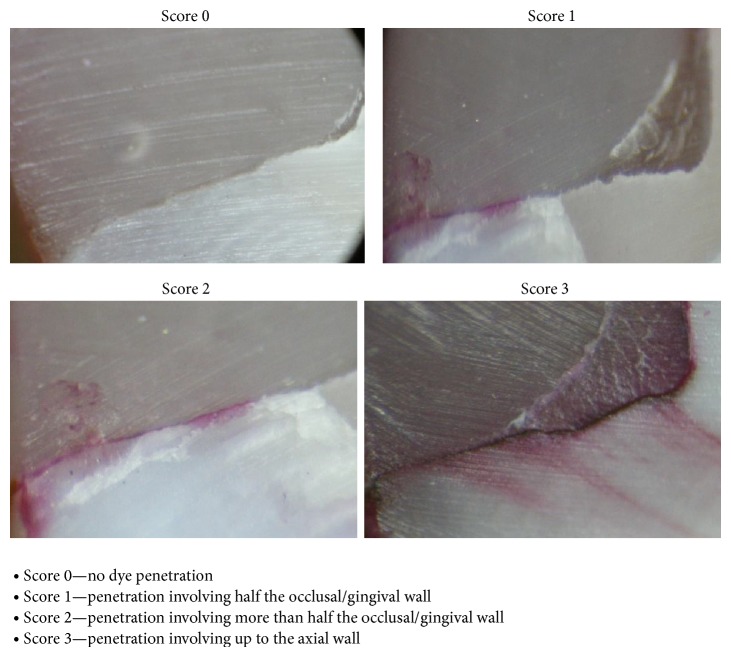


The teeth were subjected to thermocycling for 500 cycles in a water bath at 5° and 55°C with a dwelling time of 30 s after which they are subjected to cyclic loading for 10,000 cycles. Nail polish was applied to the teeth except on restorative material and tooth structure 1 mm from cavosurface margins. All specimens were immersed in 0.5% basic fuchsin solution for 24 hrs. The teeth were cut using diamond disc. Sectioned restorations were examined under a stereomicroscope at ×30 magnification. Depth of dye penetration was analyzed according to a 0–3 scale scoring system as suggested by Silveira de Araújo et al. [10] (see Figure 2).

Wilcoxon test was used to compare occlusal and gingival scores of each material. Kruskal Wallis one-way analysis of variance (ANOVA) was used to compare the occlusal and gingival scores for each group of restoration. Significance was considered at the ≤0.05 level.

## 3. Results (See Tables 1 and 2)

Significance was considered when *P* value was ≤0.05.

- The statistical analysis showed that there was no significant difference between all the materials for the occlusal margins (*P* = 0.573). But there was a very significant difference at the gingival margins (*P* = 0.004).
- Group C showed significantly less leakage than Groups A and B at gingival margins (*P* = 0.001 and *P* = 0.024). Between Groups A and B there was no significant difference (0.334).

## 4. Discussion

Because of constant increase in aesthetic demands bonded composites have been the common choice for the aesthetic restorations of class V lesions [11]. One of the main reasons for failure of composites is interfacial defects which develop as a result of long time thermal and mechanical stresses, stresses developed due to polymerisation shrinkage, and physical and chemical properties of the material. These interfacial defects can lead to microleakage which is a matter of concern because it can lead to staining at the margins of restorations, recurrent caries, hypersensitivity, and pulp pathology [12].

Microleakage is an important property that has been used in assessing the success of any restorative material used in restoring tooth [13]. Improvements in resin composites have increased their usefulness as restorative materials; however, polymerization shrinkage continues to remain one of the primary deficiencies of composite restorations. Polymerization shrinkage causes contraction stress within the restoration that leads to microleakage, as well as stress within the surrounding tooth structure. Possible reasons for microleakage at the dentin restoration margin are cavity configuration (C-factor), dentinal tubule orientation to the cervical wall (CEJ), organic content of dentine substrate and movement of dentinal tubular fluids, incomplete alteration or removal of smear layer by acidic primers (self-etch system) for adequate demineralization and hybrid layer formation, inefficient infiltration/penetration of primer components into the demineralized collagen fibrils, dentin substrates hydration level, incomplete evaporation of the solvent from the dentin surface prior to attachment of the adhesive monomers, incompatibility of the bonding agent with the respective resin composite, acid component composition (pH, osmolarity, and thickening agent), polymerization contraction, physical characteristics of the restorative material, (filler loading, volumetric expansion, and modulus of elasticity), inadequate margin adaptation of restorative material, polymerization source-photoinitiator incompatibilities and instrumentation, and finishing and polishing effects.

Hence the current study examined the microleakage of different composite resins placed in class V cavities using a dye penetration test. In the present study class V cavities are selected because cervical lesions have been a restorative challenge for any kind of restorative material due to their complex morphology where the margins are partly in enamel and partly in dentin/cementum. The primary problem associated with the restoration of class V cavities is microleakage at gingival margins located in dentin [10]. In this study dye penetration method was used because it is the most frequently used method for detecting microleakage [14]. In this study, thermocycling was done because it is a widely used method in dental research to simulate temperature changes that take place in the oral environment [15]. The cyclic loading was done in this study because occlusal stress generated in the cervical region during normal function and parafunction may increase microleakage and deteriorate the margins of class V restorations [16]. To reduce the stress magnitude in composite restoration a low stiffness material is applied between the restoration and cavity walls to increase the compliance of bonding substrate. Another benefit from this procedure is that stress distribution is more uniform along the low elastic modulus layer. This technique is called elastic cavity wall and is accomplished by the use of intermediate layer of low viscosity flowable composite which causes reduction in microleakage [17]. Simi and Suprabha showed that the marginal adaptation of a composite improved when used in conjunction with a flowable composite. Chuang et al. concluded that a 0.5–1.0 mm layer of flowable composite liner used under packable composite restorations resulted in a significant reduction in microleakage. A flowable composite was used as a liner. The injectable composite which has been recently introduced in the field of aesthetic dentistry has claimed to have a low modulus of elasticity and prepolymerised filler (organic fillers) along with inorganic fillers. The prepolymerised fillers reduce the volumetric shrinkage by increasing the available sites for composite flow without reducing the mechanical properties [18]. An injectable composite G-ænial Universal Flo was used. The results obtained in this study showed that all three composite resins that were investigated exhibited more microleakage on the gingival margins than on the occlusal margins because the flexural stresses at cervical margins are much more higher than that at the occlusal margins which is in accordance with previous studies by Nayak et al. and Kumar Gupta et al. [19, 20]. In this study Groups A (composite) and B (with flowable liner) showed high levels of dye penetration in gingival margins compared to Group C (G-ænial Flo). High flexibility, prepolymerised fillers, and low volumetric shrinkage of G-ænial Flo may be the possible reason for less microleakage compared to the other two groups.

## 5. Conclusion

Within the limitations of this study, none of the three materials were free from microleakage. All the three materials showed more microleakage at gingival margins compared to occlusal margins. Among all the groups G-ænial Flo showed the least microleakage at the gingival wall.

## Figures and Tables

**Table 1:** 

Groups	Dye leakage at occlusal margin				Dye leakage at gingival margin			
	0	1	2	3	0	1	2	3
Group A	7	7	3	3	0	3	5	12
Group B	6	12	0	2	0	2	3	15
Group C	4	10	6	0	0	3	14	3

**Table 2:** 

Groups	Occlusal	Gingival
Group A, Group B, and Group C	0.573	0.004^*^
Group A and Group B	0.64	0.334
Group B and Group C	0.731	0.024^*^
Group A and Group C	0.231	<0.001^*^

^*^Statistically significant difference.
